# Arundel Eye-Glasses

**Published:** 1877-01-20

**Authors:** 


ARUNDEL EYE-GLASSES.—Do you use eye-glasses? If so, you do not know what good eye-glasses are, if you have never used the *violet tinted Arundel.* The writer has tried them, and speaks knowingly. If you will send your number to Mr. Er Lawshe, of this city, he will fit you up and make you happy, at the small cost of $3.50 for frames and glasses. You will never regret it. G.

